# Looking Ahead to 2030: Survey of Evolving Needs in Pharmacy Education

**DOI:** 10.3390/pharmacy9010059

**Published:** 2021-03-17

**Authors:** Vassilios Papadopoulos, Dana Goldman, Clay Wang, Michele Keller, Steven Chen

**Affiliations:** 1 School of Pharmacy, University of Southern California, Los Angeles, CA 90089, USA; dpgoldman@usc.edu (D.G.); clayw@usc.edu (C.W.); kellermi@usc.edu (M.K.); 2 Sol Price School of Public Policy, University of Southern California, Los Angeles, CA 90089, USA

**Keywords:** education, forecasting, curriculum

## Abstract

In order to keep pharmacy education relevant to a rapidly-evolving future, this study sought to identify key insights from leaders from a broad array of pharmacy and non-pharmacy industries on the future of the pharmacy profession, pharmaceutical sciences, and pharmacy education. Thought leaders representing a variety of industries were surveyed regarding their perspectives on the future of pharmacy practice, pharmaceutical science disciplines, and pharmacy education in seven domains. From 46 completed surveys, top challenges/threats were barriers that limit clinical practice opportunities, excessive supply of pharmacists, and high drug costs. Major changes in the drug distribution system, automation/robotics, and new therapeutic approaches were identified as the top technological disrupters. Key drivers of pharmacy education included the primary care provider shortage, growing use of technology and data, and rising drug costs. The most significant sources of job growth outside of retail and hospital settings were managed care organizations, technology/biotech/pharmaceutical companies, and ambulatory care practices. Needs in the industry included clinical management of complex patients, leadership and management, pharmaceutical scientists, and implementation science. Knowledge gaps were pharmacists not recognizing their value on the health care team, preparation to embrace and lead change, and expertise in data science and analytics. Pharmacy schools will need to address several disruptive trends to future-proof their curricula, including expanding patient management skills, leadership and management training, technology, and data analytics.

## 1. Introduction

The U.S. healthcare system has changed dramatically over the past two decades, driven by the highest per capita cost while delivering the lowest quality of care among peer countries [1]. The rapid pace of change challenges health profession schools with ensuring their curriculum is relevant to current demands while preparing graduates for developing needs and opportunities. Projections of job growth in pharmacy suggest that the profession has not been successful in this regard. Between 2002 and 2012, the growth rate of pharmacist jobs was 20.5% compared to the national employment growth average of 14.8%; while seemingly impressive, the projected growth rate for pharmacy jobs was 30.1% [2]. Current growth projections for pharmacy jobs between 2016 and 2026 is 5.6%, which is 25% lower than the projected national employment growth of 7.4% [3]. In addition, according to the U.S Bureau of Labor Statistics, job growth for pharmacists from 2018 to 2028 is projected to be 0%, with a national employment growth average of 5% [4]. These projections suggest a misalignment between how pharmacy schools are preparing graduates and market needs/demand.

In the past 10 years, the number of pharmacy schools in the U.S. increased 41% from 100 to 141, PharmD student enrollment increased 25% from 50,691 to 63,464, and annual PharmD graduates rose 48% from 9812 to 14,556. Based on these rates, over the next 10 years, it is estimated that ~145,000 new pharmacists will be seeking to replace nearly half of the currently employed pharmacists [5]. While supply has clearly increased, demand has not kept up. A multitude of factors accounts for the declining job opportunities in pharmacy. These include a decrease in the number of retail pharmacies and mergers between chain pharmacies and health systems/pharmacy benefit management companies; expanded use of technology that reduces the number of pharmacists needed for technical functions; slower expansion of clinical practice opportunities than anticipated; and delayed retirements combined with an oversupply of pharmacy graduates [6,7].

Pharmacy practice and education have continually evolved, but most changes have been subtle and not consistently informed by data or forecasts outside of the profession, leading to a lack of current professional identity and an unclear path for the future [8]. In its 2019–2020 report, the Argus Commission referred to the importance of routinely monitoring changes in the healthcare field to the academic pharmacy. The scope of vigilance and its influence should extend beyond didactic courses, reaching the broader spectrum of activities that students can utilize to advance their skills and knowledge, including organizational activities and portfolios [9]. The American Society of Health-System Pharmacists conducts a comprehensive Pharmacy Forecast each year, providing insight into emerging trends that impact pharmacy practice [10]. Survey participants for the Pharmacy Forecast are stringently vetted and highly accomplished but include only leaders within the pharmacy profession.

While pharmacy schools have implemented PharmD curricula that meet the ACPE Standards, the transformation of the profession to more patient-care focused roles envisioned by academia has not materialized at the same rate as the supply of new pharmacists. In addition, the healthcare system has evolved rapidly, particularly over the past decade, making it a challenge to maintain a curriculum that prepares graduates for future practice. In addition, as COVID-19 has demonstrated, a pandemic can completely upend the most well-developed teaching strategy, requiring rapid and thoughtful responses and adaptations from schools and accrediting agencies. As a result, associations and leaders within the academy must lead disruptive change in pharmacists’ roles beginning within the pharmacy curriculum. The purpose of this paper is to share details and results of a survey completed by thought leaders within and outside of the pharmacy industry that provided insights into the future of the pharmacy profession and pharmaceutical sciences discipline. By adding experts outside of pharmacy, we hope to gain new insights to help inform strategic planning for pharmacy education.

## 2. Materials and Methods

The USC School of Pharmacy collaborated with an external consultant, AMC Strategies, to conduct this survey. AMC Strategies was chosen because of its 20-year history of helping leading healthcare and academic health institutions develop strategic planning solutions. Clients include Baylor, Columbia, Duke, Georgetown, University of Utah, and University of Virginia. AMC Strategies’ understanding of the California landscape through clients such as Stanford, Cedars-Sinai, City of Hope, and a half dozen University of California schools added to our interest in collaborating. Senior leadership at the USC School of Pharmacy met with AMC Strategies consultants to select thought leaders representing pharmacy and non-pharmacy industries, including experts in health systems, government healthcare, pharmacy supply chain, independent pharmacy, academia, pharmaceutical industry, pharmacy professional organizations, and venture capital. Twenty-seven senior leaders were identified based on national or international influence and reputation. In addition, 19 USC School of Pharmacy faculty and members of the board of councilors representing a broad range of expertise were included for a total 46 participants. Although the focus of the survey was on external leaders, many faculty and board of councilor members have national or international influence and represent a broad range of industries within and outside of pharmacy. Sixteen faculty, residents, and students were selected representing leaders of departments, education (PharmD students, residents/fellows, and graduate students), development, and communications to serve on the steering committee. Guided by the external consultant, the steering committee developed with following open-ended questions to solicit insights into the future of pharmacy and pharmacy education:

1. Challenges/Threats: What are the greatest challenges, or threats, facing pharmacy practice and the pharmaceutical industry over the next 10 to 20 years?
2. Innovation/Technology: What groundbreaking innovation and technology could completely disrupt pharmacy practice and the pharmaceutical industry in the next 10–20 years?
3. Key Trends/How to Prepare: What are the key trends that may drive pharmacy education and training? What should be done to prepare future pharmacists/pharmaceutical scientists to meet these trends?
4. Job Growth: Where do you anticipate the greatest source of job growth for pharmacists outside of retail and hospital settings?
5. Industry-specific Needs: What needs do you anticipate for pharmacists and/or pharmaceutical scientists in your industry?
6. Gaps in Knowledge/Wish They Knew: What do you wish your pharmacists/pharmaceutical scientists knew that they do not know now?
7. Critical for Future Preparation: In summary, describe three aspects of pharmacy education and training that will be most critical in preparing pharmacists and pharmaceutical scientists for the future.

The survey was administered as quantitatively based structured interviews conducted one-on-one with participants by the consultant group, which included open-ended questions. The analysis of results was quantitative, limited to descriptive statistics and frequency counts due to the small sample size.

## 3. Results

All 46 participants completed the survey. The responses shared were aggregated from open-ended prompts, with the percentages reflecting the proportion of survey findings where a particular topic was mentioned.

The top challenges or threats identified facing pharmacy practice and the pharmaceutical industry over the next 10 to 20 years included barriers that limit clinical practice opportunities, with emphasis on the lack of sustainable compensation models, inconsistency in licensing/credentialing practices, and a general culture of medicine that is often resistant to pharmacy policy and practice changes attempting to expand scope or payment opportunities. An excessive supply of pharmacists relative to demand was the second most significant challenge, attributed at least in part to changes in the medication distribution and dispensing process and technology adoption that reduce the number of pharmacists needed, and the increase in the number of pharmacy schools and pharmacy graduates. Several other challenges and threats were identified, including the failure of pharmacy schools to modify education and training to meet future needs (Table 1).

Several of the top groundbreaking innovations or technologies identified that could disrupt pharmacy practice and the pharmaceutical industry in the next 10 to 20 years centered on artificial intelligence, data analytics, and technology-enhanced patient care management. Thought leaders believed use of artificial intelligence and predictive analytics for managing drug dosing, contraindications, and adverse effects would expand, along with technology-enhanced Comprehensive Medication Management and monitoring (including telehealth, wearable monitoring devices, and machine learning integration into medical records), and continued health data digitization and systematic use of data analytics. Other innovations include major changes in the drug distribution system such as online and mail-order pharmacy, consolidation in retail pharmacy chains, and closure of community pharmacies. Almost one-third of respondents agreed that new therapeutic approaches such as alternative modalities for administering medications and cellular and genetic therapies would disrupt pharmacy. These and other responses are summarized in Table 2.

At least one-third of respondents identified the following as significant influences on pharmacy education and training: the primary care provider shortage, expanding use of technology and data, rising drug costs, growing regulatory oversight, innovation in drug development and distribution, and the shift towards value-based care. Other influences are listed in Table 3.

In anticipating the greatest sources of job growth outside of retail and hospital settings, respondents identified managed care organizations, technology companies, biotech, and pharmaceutical companies, ambulatory care practices, online pharmacies, government, health insurance companies, and pharmacy benefits management as the major industries. Occupations or skills commensurate with these industries included data science, Comprehensive Medication Management, operations, specialty and primary care, regulatory management, public health, and telehealth.

When considering anticipated needs for pharmacists and/or pharmaceutical scientists in their own industry, over one-fifth of respondents identified clinical management of complex patients and leadership/management skills. Between 10 and 20% of respondents recognized pharmaceutical science, evaluation and implementation science, and pharmacy school faculty as needs, although one respondent believes there will be a surplus of faculty resulting from the downsizing of PharmD programs through lower enrollment numbers and/or closures of pharmacy schools. Health economics and policy, public health expertise, and data science were considered by less than 10% of our experts.

In regards to knowledge gaps among pharmacists and pharmaceutical scientists (Table 4), interestingly, almost one-third of respondents believed that pharmacists lack awareness of their unique value and impact on patient health outcomes as a member of the healthcare team. One-quarter of respondents suggested that pharmacists are unprepared to embrace and lead change. At the same time, just over one in five indicated that pharmacists underrecognize the need for data science and analytics expertise.

**Figure 1:**
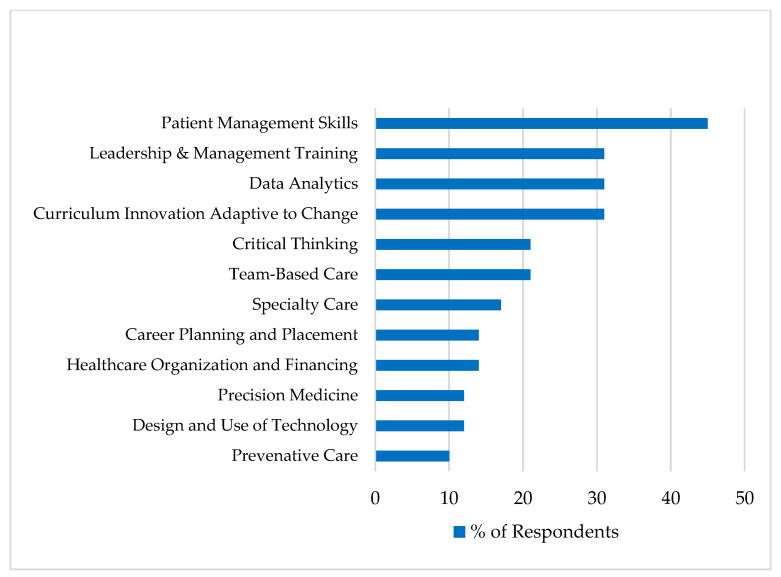
Aspects of pharmacy education and training most critical in preparing pharmacists and pharmaceutical scientists for the future.

When considering the most critical aspects of pharmacy education and training to prepare graduates for the future, patient management skills stood out by far, identified by nearly half of all respondents. Nearly one in three mentioned leadership and management training, data analytics, and curriculum innovation that adapts to a dynamic future. Other considerations are summarized in Figure 1.

## 4. Discussion

Reponses to our survey provided useful information for pharmacy education strategy, with critical input from experts outside of pharmacy. Drug costs were a common concern, leading to the importance of training students on health economics, regulatory sciences, and managing expensive and complex medication therapies. Advancements and disruptions in drug development, distribution, and dispensing require students to have skills in data analysis, systems design and machine learning, clinical research, supply chain management, and the business of healthcare and pharmacy. Healthcare financing and payment for clinical services is a major knowledge gap for most pharmacy students. However, the future of clinical pharmacy practice is dependent on sustainable payments for these services. Heavy emphasis was placed on developing excellent clinical practice pharmacists with skills in differential diagnosis, Comprehensive Medication Management (which encompasses patient safety along with treatment efficacy), enhanced critical thinking skills, communication and leadership excellence, early exposure to patient care and complex care management, team-based care/interprofessional education, and specialty training. With tremendous disparities among various populations, social determinants of health, health economics, and public health, and curating and recruiting diverse learners are a high priority. Combining clinical practice excellence and health disparities/access can be addressed by deploying community pharmacists as neighborhood hubs for health and social services, including Comprehensive Medication Management. The rapid pace of data and technological advances warrants training in the collection, management analysis, and dissemination of data and continuing development of digital wearables, telehealth, and access to electronic health records/health information exchanges to ensure optimal and efficient medication safety and efficacy. Finally, the expansion of personalized medicine through compounded and/or customized dosing and delivery mechanisms, pharmacogenomics, genetics, biostatistics, and bioengineering are important areas of emphasis for developing pharmacists and pharmaceutical scientists.

The findings from this survey are consistent with several others but offer broader insights from leaders in multiple industries [11,12]. Given the multitude of needs identified in pharmacy education, a major challenge for pharmacy schools is adding new curricular content when the existing curriculum is already full, with few areas that can be removed or further condensed. Instead, schools should consider tailoring learning for students by helping them identify career paths aligned with different tracks while continuing to provide training on core materials/topics to all students. In addition, revision of ACPE accreditation standards to support a more adaptable curriculum and alignment with post-graduate training (residencies and fellowships) will maximize educational efficiency and quality.

The arrival of COVID-19 in 2020 has accelerated some of the changes in the pharmacy environment. These were led by the increased use of telehealth and increase in the scope of practice of pharmacists in almost all states across the nation. Moreover, the accelerated need for drug discovery, vaccine development, accelerated clinical trials, and regulatory pathways for FDA approvals needed to battle the disease has increased the need for pharmacists and pharmaceutical scientists with the skills and knowledge identified above.

Several limitations of this survey should be noted. The sample size for a survey was small, limiting statistics to descriptive. As a result, the findings can still be valuable but are not generalizable; recommendations must be offered with caveats that these results are from a particular sample and more research needs to be done on a larger scale. In addition, while we tried to represent a broad array of disciplines and industries, all of the multiple market influences that drive healthcare and medication costs, incentive behaviors, decrease reimbursements, limit payor models across all states, and produce sustainable implementation of disruptive technologies could not be included. Lastly, the inclusion of some faculty may be viewed as contrary to the goal of seeking external perspectives; however, participation was limited to select faculty members whose work or partnerships have statewide or national influence.

## 5. Conclusions

As with many health profession schools, academic pharmacy is challenged with offering a dynamic curriculum that meets current and evolving needs in healthcare. By soliciting perspectives from a broad group of stakeholders, with more than a dozen from industries and expertise outside of pharmacy, we were able to gain insights that can inform the design of pharmacy school educational strategies. Although some feedback simply confirmed the trajectory of most schools, many suggestions challenge the current course of pharmacy education. For the practice of pharmacy, a major theme is that medication management is an essential component of delivering high-quality healthcare, and patients with complex health and medication therapies need to be managed outside of the physician’s office. Pharmacy education must prepare pharmacists for this model of care.

Unprecedented challenges facing pharmacy today, including the projected lack of job growth and challenges and opportunities brought on by the COVID-19 pandemic, warrant a disruptive re-evaluation of pharmacy education. Changes in direction should be considered together with all pharmacy stakeholders: schools of pharmacy, post-graduate training programs, pharmacy associations, and employers, as well as pharmacy technician organizations and schools. If pharmacy schools provide education on sustainable solutions to real-world problems, students seeking to join the profession of pharmacy will be prepared to embrace the unknown and contribute to important societal needs. Schools of pharmacy are obligated to prepare graduates for a future that realizes the full potential of their education and training, as well as shapes the future of pharmacy practice through scholarship, partnerships, policy, and innovation that addresses critical healthcare needs in a sustainable and scalable approach.

## Figures and Tables

**Table 1:** Responses to the question, “What are the greatest challenges, or threats, facing pharmacy practice and the pharmaceutical industry over the next 10 to 20 years?” (*n* = 46).

Response	% of Respondents
Barriers that limit clinical practice opportunities for pharmacists.	54%
Excess supply of pharmacists relative to demand.	37%
Unsustainably high drug costs limit access and compliance.	32%
Highly complex medication regimens requiring significant patient support.	24%
New technology changing the role of pharmacists in drug dispensing and patient consultation.	24%
Failure of pharmacy schools to modify education and training to meet future needs.	23%
Consolidation in health services organizations driving changes in delivery and compensation models.	22%
Limited ability to ensure safety, quality, and efficacy of pharmaceuticals.	15%
Pharmaceutical industry business model favors development of highly specialized drugs that benefit small subsets of the population.	12%

**Table 2:** Responses to the Question, “What groundbreaking innovation and technology that could completely disrupt pharmacy practice and the pharmaceutical industry in the next 10–20 years?” (*n* = 46).

Response	% of Respondents
Major changes in the drug distribution system.	39%
Use of automation and robotics in drug dispensing.	29%
New therapeutic approaches.	29%
Artificial intelligence (A.I.) and predictive analytics.	27%
Personalized medicine and micro customization of therapeutics.	24%
New models of primary care that include pharmacists on the care team.	20%
Technology for Comprehensive Medication Management & monitoring.	17%
Continued digitization of health data and systematic use of advanced data analytics.	17%

**Table 3:** Responses to the Question, “What are the key trends that may drive pharmacy education and training?” (*n* = 46).

Response	% of Respondents
Primary care provider shortage	43%
Growth in the Use of technology and data	43%
Raising drug costs	40%
Growing regulatory oversight	40%
Innovation in drug development and distribution processes	35%
Shift to value-based care	33%
Chronic disease, mental illness, and addiction	28%
Need to address social determinants of health	25%
Consumerism and patient-driven care	23%
Personalized medicine and tailored drug therapies	15%

**Table 4:** Responses to the Question, “What do you wish your pharmacists/pharmaceutical scientists knew that they do not know now?” (*n* = 46).

Response	% of Respondents
Pharmacists are valuable members of the health care team	31%
Embrace and lead change	25%
Data science and analytics	22%
Personalized medicine	17%
Strong basic science	11%
Safety, efficacy, and cost effectiveness of drug therapies	11%
Importance of social determinants of health	11%
Strong business sense	8%

## Data Availability

As a consequence of conducting structured interviews using open-ended questions, all relevant data is contained within this article.
